# Comment on Roelofs, K.A.; et al. Detecting Progression of Melanocytic Choroidal Tumors by Sequential Imaging: Is Ultrasonography Necessary? *Cancers* 2020, *12*, 1856

**DOI:** 10.3390/cancers13061306

**Published:** 2021-03-15

**Authors:** Luigi Capasso, Marco Gioia, Maddalena De Bernardo, Nicola Rosa

**Affiliations:** 1 Cornea Transplant Unit, ASL NA 1, 80137 Naples, Italy; luigicapasso@hotmail.com; 2 Department of Medicine, Surgery and Dentistry “Scuola Medica Salernitana”, University of Salerno, 84081 Baronissi, Italy; marco.gioia@yahoo.it (M.G.); nrosa@unisa.it (N.R.)

We read with great interest the paper by Roelofs et al. [1], dealing with multimodal ocular imaging in the case of choroidal nevi, to detect the progression of choroidal melanocytic tumors. However, we would like to discuss some points in the study that, in our opinion, could be misleading.

The authors utilized ultrasonography (US) solely to evaluate the mushroom shape and the growth in thickness, but they neglected to evaluate two very important echographic findings: the internal reflectivity and the detection of internal vascularity. These US parameters, detected with standardized A scan echography which was first described by KC Ossoinig in the 1970s, are very important signs for the diagnosis of melanoma and should be evaluated before the thickness increase [2].

The presence of a mushroom shape alone (due to the Bruch’s membrane rupture) should be considered an indication of probable melanoma and not simply a high-risk nevus, as suggested in Table 1 and Table 2. Unfortunately, this sign is mainly present in large melanomas, and it is very rare in small melanomas. For this reason, in the latter case, the mushroom shape is less sensitive compared to the detection of an homogeneous low–medium reflectivity, with signs of internal vascularity detectable with standardized A scan technique. Regrettably, the mushroom shape and not these signs, diagnostic for melanoma, were taken into account by the authors. The mushroom shape, due to the Bruch’s membrane rupture, in our opinion, is less important than the internal reflectivity, because it is present in large melanomas but is very rare in small/ medium size melanomas, where the low reflectivity allows a diagnosis to be made. We agree with the authors that in lesions less than 1.5 mm, Optical Coherence Tomography (OCT) might be more sensitive in detecting growth of the lesions, but, as the authors correctly stated, it is less sensitive in detecting changes in the thickness when the lesion is thicker than 2 mm; therefore, this is another important aspect for ultrasound examination, as the guidelines in the literature suggest that tumors be treated when they are more than 2 mm in thickness [3], but this quality could be missed if OCT alone is used.

The authors stated that in case 61, with initial MOLES Score = 3, the lesion was diagnosed by pointing out a sub-retinal fluid visible on OCT [4], together with an increase in thickness of 0.5 mm detected by US, and concluded that as OCT allowed the detection of tumor progression in the only case without lateral extension on color photography, progression would have been detected without ultrasonography in all 36 (100%) tumors. Therefore, as both US and OCT showed signs of progression, we could also conclude that progression would have been detected without OCT in all 36 (100%) tumors. 

Regarding patients classified as MOLES 4, among the 35 patients whose progression was detected, US alone showed a tumor increase in patient 81. This progression (3%) would have been missed without US.

Moreover, considering that all patients underwent US too, it could be suggested that the obtained outcomes were affected by bias towards determining the results of this imaging technique. Would the authors feel it is truly safe to follow high-risk nevi or probable melanoma without ultrasound imaging? 

In conclusion, we agree that in the case of lesions smaller than 1 mm, US is not useful, because with the routinely used US frequencies, the tumors are almost invisible. However, the detection of such small lesions is not particularly advantageous. In fact, the Collaborative Ocular Melanoma Study (COMS) guidelines [5] suggest that tumors be treated when they are larger than 3 mm. In addition, in the review by Chien et al. [6], among the other risk factors, a thickness of more than 2 mm is described, and this size is too thick for OCT, as the authors correctly stated. For all these reasons, in our opinion, it is highly dangerous to state that it is unnecessary to follow-up these tumors with ultrasound.

## References

[1] Roelofs K.A., O’Day R., Al Harby L., Hay G., Arora A.K., Cohen V.M.L., Sagoo M.S., Damato B.E. (2020). Detecting Progression of Melanocytic Choroidal Tumors by Sequential Imaging: Is Ultrasonography Necessary?. Cancers.

[2] Ossoinig K.C., Bigar F., Kaefring S.L. (1975). Malignant melanoma of the choroid and ciliary body. A differential diagnosis in clinical echography. Bibl. Ophthal..

[3] Jouhi S., Jager M.J., de Geus S.J.R., Desjardins L., Eide N.A., Grange J.-D., Kiilgaard J.F., Seregard S., Midena E., Parrozzani R. (2019). The Small Fatal Choroidal Melanoma Study. A Survey by the European Ophthalmic Oncology Group. Am. J. Ophthalmol..

[4] Shields C.L., Dalvin L.A., Ancona-Lezama D., Yu M.D., Di Nicola M., Williams B.K., Lucio-Alvarez J.A., Ang S.M., Maloney S., Welch R.J. (2019). Choroidal Nevus Imaging Features In 3,806 Cases And Risk Factors For Transformation Into Melanoma In 2,355 Cases: The 2020 Taylor R. Smith and Victor T. Curtin Lecture. Retina.

[5] Collaborative Ocular Melanoma Study Group (1997). Mortality in patients with small choroidal melanoma. COMS report no. 4. Arch. Ophthalmol..

[6] Chien J.L., Sioufi K., Surakiatchanukul T., Shields J.A., Shields C.L. (2017). Choroidal nevus: A review of prevalence, features, genetics, risks, and outcomes. Curr. Opin. Ophthalmol..

